# Diagnostic Accuracy and Prognostic Value of Neutrophil-to-Lymphocyte and Platelet-to-Lymphocyte Ratios in Septic Patients outside the Intensive Care Unit

**DOI:** 10.3390/medicina57080811

**Published:** 2021-08-07

**Authors:** Silvia Spoto, Domenica Marika Lupoi, Emanuele Valeriani, Marta Fogolari, Luciana Locorriere, Giuseppina Beretta Anguissola, Giulia Battifoglia, Damiano Caputo, Alessandro Coppola, Sebastiano Costantino, Massimo Ciccozzi, Silvia Angeletti

**Affiliations:** 1 Diagnostic and Therapeutic Medicine Department, University Campus Bio-Medico of Rome, 00128 Roma, Italy; s.spoto@unicampus.it (S.S.); domenicamarika.lupoi@unicampus.it (D.M.L.); e.valeriani@unicampus.it (E.V.); l.locorriere@unicampus.it (L.L.); g.beretta@unicampus.it (G.B.A.); g.battifogliaa@gmail.com (G.B.); s.costantino@unicampus.it (S.C.); 2 Unit of Clinical Laboratory Science, University Campus Bio-Medico of Rome, 00128 Roma, Italy; s.angeletti@unicampus.it; 3 Department of Surgery, University Campus Bio-Medico of Rome, 00128 Roma, Italy; d.caputo@unicampus.it (D.C.); a.coppola@unicampus.it (A.C.); 4 Unit of Medical Statistics and Molecular Epidemiology, University Campus Bio-Medico of Rome, 00128 Roma, Italy; m.ciccozzi@unicampus.it

**Keywords:** neutrophil-to-lymphocyte, platelet-to-lymphocyte, C-reactive protein, procalcitonin, MRproAdrenomedullin, systemic inflammatory response syndrome, sequential organ failure assessment, quick-sequential organ failure assessment, sepsis, septic shock

## Abstract

*Background and Objectives*: The aim of this study was to evaluate the diagnostic accuracy and prognostic value of neutrophil-to-lymphocyte (NLR) and platelet-to-lymphocyte (PLR) ratios and to compare them with other biomarkers and clinical scores of sepsis outside the intensive care unit. *Materials and methods*: In this retrospective study, 251 patients with sepsis and 126 patients with infection other than sepsis were enrolled. NLR and PLR were calculated as the ratio between absolute values of neutrophils, lymphocytes, and platelets by complete blood counts performed on whole blood by *Sysmex* XE-9000 (Dasit, Italy) following the manufacturer’s instruction. *Results*: The best NLR value in diagnosis of sepsis was 7.97 with sensibility, specificity, AUC, PPV, and NPV of 64.26%, 80.16%, 0.74 (*p* < 0.001), 86.49%, and 53.18%, respectively. The diagnostic role of NLR significantly increases when PLR, C-reactive protein (PCR), procalcitonin (PCT), and mid-regional pro-adrenomedullin (MR-proADM) values, as well as systemic inflammatory re-sponse syndrome (SIRS), sequential organ failure assessment (SOFA), and quick-sequential organ failure assessment (qSOFA) scores, were added to the model. The best value of NLR in predicting 90-day mortality was 9.05 with sensibility, specificity, AUC, PPV, and NPV of 69.57%, 61.44%, 0.66 (*p* < 0.0001), 28.9%, and 89.9%, respectively. Sensibility, specificity, AUC, PPV, and NPV of NLR increase if PLR, PCR, PCT, MR-proADM, SIRS, qSOFA, and SOFA scores are added to NLR. *Conclusions*: NLR and PLR represent a widely useful and cheap tool in diagnosis and in predict-ing 90-day mortality in patients with sepsis.

## 1. Introduction

Sepsis is a systemic syndrome induced by infection and leading to a widespread inflammation up to septic shock, multi organ failure, and death [1,2]. Patients with bacteriemia, sepsis, and septic shock presented a high mortality rate ranging from 25% to 30% and 40% to 50%, respectively [3,4]. Patients’ prognosis and mortality rate, however, are strictly affected by a timely performed clinical and laboratory diagnosis as well as by proper therapeutic management [5,6,7].

Blood cultures represent the gold standard for microbiological diagnosis of sepsis [6]. Unfortunately, they yielded positive results in just a third of cases and may require several days for positivization even if newer and more expensive molecular techniques are used (e.g., polymerase chain reaction and mass spectroscopy) [8,9,10,11,12,13,14,15,16,17,18].

To overcome these issues, several scores such as SIRS and qSOFA were introduced in clinical practice to help diagnosis, disease severity stratification, and prognostic evaluation [5,19,20]. Adding laboratory biomarkers increases the usefulness of these scores in guiding clinical and therapeutic choices [12,13,14,15,16,17,18,19,20,21,22,23,24,25,26]. Among these, C-reactive protein (PCR, ≥ 5 mg/dL), procalcitonin (PCT, ≥0.5 ng/mL), and mid-regional pro-adrenomedullin (MR-proADM, ≥1.50 nmol/L) showed the highest diagnostic and prognostic power, but they were expensive and not widely available [15,26,27,28,29]. Conversely, the neutrophil-to-lymphocyte ratio (NLR) represents a widely available, inexpensive, and easily performed marker that has been recently evaluated for its diagnostic and prognostic role in sepsis. NLR early expresses the relationship between innate (neutrophils) and adaptive cellular immune response (lymphocytes) during pathological states. [30]. Mean NLR values below 2 (1,6) are representative of healthy people (without differences in sex category or race) [30,31], while it may increase up to values of >10 in sepsis and >20 in septic shock, with good sensibility and specificity [30,31,32,33,34,35,36,37,38,39,40,41,42,43,44]. NLR seems to also vary in relation to different bacterial pathogens, with the lowest and highest values in Gram-positive and Gram-negative or polymicrobial sepsis, respectively [37,38,39].

NLR, however, may be affected by some clinical condition or therapies resulting in false positive (e.g., corticosteroids) or false negative (e.g., chemotherapy, radiotherapy, antibiotic therapy, Cachexia) results [31].

Along with NLR, the monocyte-to-lymphocyte ratio (MLR), the platelet-to-lymphocyte ratio (PLR), and the mean platelet volume-to-platelet count (MPV/PC) ratio have been studied recently, but the results are contrasting [30,31,32,33,34,35,36,37,38,39].

The aim of this study was to evaluate the diagnostic accuracy and prognostic value of NLR, PLR, and MLR in patients with sepsis and septic shock outside the intensive care unit (ICU) and to compare them with C-reactive protein (CRP), PCT, MR-proADM, *SIRS*, qSOFA, and SOFA scores.

Furthermore, we evaluated the role of NLR in aetiological diagnosis of sepsis and on length of stay stratification.

## 2. Materials and Methods

The study was approved on 23 July 2016 by the Ethical Committee of the University Hospital Campus Bio-Medico of Rome (28.16 TS Com Et CBM). All methods were performed in accordance with the relevant guidelines and regulations. Informed consent was not required for the retrospective design of the study.

### 2.1. Patients Selection and Study Design

Consecutive patients with clinically suspected sepsis or septic shock admitted to the Diagnostic and Therapeutic Medicine Department and General Surgery of the University Hospital Campus Bio-Medico of Rome were retrospectively enrolled between May 2014 and February 2021.

Exclusion criteria were age < 18 years and pregnancy.

The control group included patients with infection, but without sepsis admitted to the Diagnostic and Therapeutic Medicine Department between May 2014 and February 2021.

Diagnosis of sepsis was performed according to the Third Consensus Conference Criteria of 2016 when qSOFA or SOFA scores were ≥2 from the baseline in the presence of an infection.

Bloodstream infection was defined as any positive blood culture for pathogens. Pneumonia was defined based on a positive pathogen respiratory culture and other Infectious Diseases Society of America (IDSA) diagnostic criteria [45]. Patients with positive urine cultures were identified as cases based on the CDC National Healthcare Safety Network (NHSN) UTI case definitions [46].

Baseline patients’ characteristics were retrospectively collected form medical records including demographic information (age, sex category), presence of comorbidities (cardiovascular, pulmonary, kidney, liver disease), immune status (active malignancy or other causes of an immunosuppression), immunosuppressive treatments (corticosteroids, antibiotics), laboratory values (complete blood count, NLR, PLR, MLR, PCR, PCT, MR-proADM), and clinical scores (e.g., SIRS, qSOFA, SOFA).

### 2.2. Laboratory and Microbiological Parameters

Complete blood counts (CBCs) were performed on whole blood by *Sysmex* XE-9000 (Dasit, Italy) following the manufacturer’s instruction. NLR, PLR, and MLR were calculated by the ratio between absolute values of neutrophils, lymphocytes, monocytes, respectively, and that of platelets.

CRP protein was measured by Alinity c (Abbott, diagnostics) following the manufacturer’s instruction.

PCT and MR-proADM plasma concentrations were measured by an automated Kryptor analyzer, using a time-resolved amplified cryptate emission (TRACE) technology assay (Kryptor PCT; Brahms AG; Hennigsdorf, Germany) with commercially available immunoluminometric assays (Brahms) [5,21,25,26].

Blood specimens from patients were collected in BACTEC bottles containing anaerobic or aerobic broth and resins. Blood culture bottles were incubated in BACTEC FX instrument (Becton Dickinson, Meylan, France) until they were positive for bacterial growth or for a maximum of 5 days. Positive samples were cultivated in selective agar media. Growing colonies were identified by MALDI-TOF (Brahms) [5,21,25,26]. Selective and non-selective media were used for microbiological cultures.

### 2.3. Statistical Analysis

Data were analysed using Med-Calc 11.6.1.0 statistical package (MedCalc Software, Mariakerke, Belgium). Receiver operating characteristic (ROC) analysis was performed among independent variables associated with sepsis to define the cutoff point for NLR, PLR, plasma PCR, PCT, MR-proADM, SIRS, SOFA, and qSOFA score values. ROC curves and areas under the curve (AUCs) were calculated for all markers and compared in patients with sepsis or septic shock versus control patients.

χ2 for proportions test was used to compare the relative percentage of patients with positivity and/or negativity to SIRS criteria, SOFA score, qSOFA score, and other demographic characteristics of septic patients and control patients.

Positive predictive value (PPV) and negative predictive value (NPV) were calculated for each variable, based on sensitivity, specificity, and disease prevalence. Younden Index was used for cut-off selection.

The multivariate logistic regression model is performed to evaluate the association between all evaluable laboratory markers and 90-day mortality.

Mann–Whitney test was used for median values’ comparison. *p*-value < 0.05 was considered significant.

## 3. Results

### 3.1. Baseline Patients’ Characteristics

Demographic and clinical characteristics of patients with sepsis (251 patients) and the control group (126 patients) are reported in Table 1.

Patients with sepsis were younger than the control group (73 vs. 80, *p* = 0.001), while roughly half of the patients in both groups were male (52.6 vs. 50.4%, *p* = 0.771).

The vast majority of baseline patients’ characteristics were similar between septic patients and control group (Table 1), except for the presence of presence of cancer and chronic lung disease that was more (36.7% vs. 23.8%, *p* = 0.016) and less frequent (23.1 vs. 34.1, *p* = 0.031), respectively, in the former.

In septic patients, median SIRS, qSOFA, and SOFA scores’ values were 2 (IQR, 1 to 3), 2 (IQR, 1 to 2), and 4 (IQR, 2 to 6), respectively. One hundred out of 251 patients (39.8%) had septic shock and 47 out of 251 patients (18.7%) required ICU transfer during hospitalization.

The median length of stay was higher in septic patients than the control group (15 days (IQR, 11 to 26) vs. 10 days (IQR 7 to 13), *p* ≤ 0.001) and a significantly higher proportion of patients with sepsis died during 90-day follow-up (27.5% vs. 0.8%, *p* < 0.001).

### 3.2. Diagnostic Role of NLR

**Figure 1:**
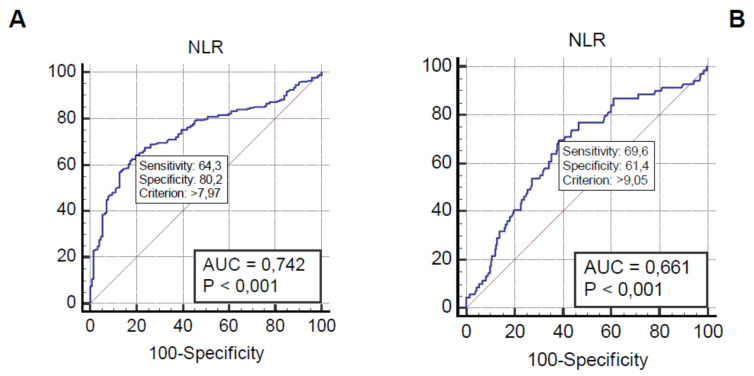
(**A**) Receiver operating characteristic (ROC) curve analysis, showing neutrophil-to-lymphocyte (NLR) ability to differentiate Scheme 7.97. (**B**) ROC curve analysis, showing NLR ability in to predict 90-day mortality in septic patients; the best value of NRL was 9.05.

For the diagnosis of sepsis, the best value of NLR was 7.97 with sensibility of 64.26%, specificity of 80.16%, AUC of 0.74 (*p* < 0.001), PPV of 86.49%, and NPV of 53.18%. The ROC curve is reported in Figure 1A. In Table 2, the diagnostic role of NLR is compared with that of PLR, PCR, PCT, and MR-proADM, as well as with that of SIRS, q-SOFA, and SOFA scores. MLR did not reach a significant role in the diagnosis of sepsis.

The diagnostic role of NLR significantly increases when PCR, PCT, and MR-proADM values, as well as SIRS, qSOFA, and SOFA scores, were added to the model (Table 3).

When just PCT and MR-proADM are considered in the diagnosis of sepsis, the model reached a PPV of 96% and a NPV of 69%. PPV and NPV for SIRS ≥2, qSOFA ≥2, and SOFA ≥2 were 96.77% and 43.3%, 99.23 and 50.21%, and 82.94% and 54.21%, respectively.

The best values of PPV and NPV are reached when NLR, PLR, and SIRS scores (99.7% and 94%, respectively), or NLR, PLR, and qSOFA scores (99.9% and 95.6%, respectively), are included in the model.

NLR, MLR, and PLR did not show a significant role in aetiological diagnosis of sepsis. Conversely, our results confirm the role of PCT in aetiological diagnosis of sepsis with higher values in Gram-negative versus Gram-positive bacteria (*p* = 0.0022). Furthermore, MR-proADM values are significantly higher in Gram-negative (*p* = 0.037) and polymicrobial (*p* = 0.037) than Gram-positive sepsis.

### 3.3. Role of NLR in Predicting 90-Day Mortality

The best value of NLR in predicting 90-day mortality was 9.05, with sensibility, specificity, AUC, PPV, and NPV of 69.57%, 61.44%, 0.66 (*p* < 0.0001), 28.9%, and 89.9%, respectively. The ROC curve is reported in Figure 1B.

The prognostic role of NLR in comparison with that of PLR, PCR, PCT, and MR-proADM values, as well as with that of SIRS, qSOFA, and SOFA scores, is listed in Table 4.

Sensibility, specificity, AUC, PPV, and NPV of NLR increase if PLR, PCR, PCT, MR-proADM, SIRS, qSOFA, and SOFA scores are added to NLR (Table 5).

MLR was not statistically significant in the 90-day mortality prediction.

Multivariate logistic regression model including all evaluable laboratory markers showed as just MR-proADM is significantly associated with 90-day mortality (Table 6).

## 4. Discussion

The results of this study showed that NLR values of 7.97 had a good diagnostic accuracy, whereas a value of 9.05 allowed a prognostic stratification of patients with sepsis that is increased by the association with PLR values of 370.59. Conversely to PCT and MR-proADM, NLR did not help identify the type of bacterial pathogen responsible for sepsis. MLR evaluation did not yield significant results.

Patients with sepsis presented a higher 90-day mortality (27.5%) and need for ICU transfer (18.7%) than the control group. However, these proportions of patients resulted lower than data available from previous studies, where mortality and ICU transfer reached values as high as 37.5% and 80.8%, respectively [47].

Performing a complete blood count and calculating NLR and PLR in a clinical suspicion of sepsis may, therefore, help the clinician in diagnostic evaluation and prognostic stratification of patients with significant values of sensibility, specificity, PPV, NPV, and AUC (*p* < 0.0001). These latter values were similar to the values of PCT >0.41 and MR-proADM >1.83 and are increased by the association with PLR values (PPV of 96% and NPV of 83%) and clinical score of sepsis such as SIRS (PPV of 99.7% and NPV of 94.0%), qSOFA (PPV of 99.9% and NPV of 95.6%), and SOFA (PPV 98.3% and NPV on 91.6%). In our study, the association between NLR and SIRS or qSOFA reached higher diagnostic power than the association between NLR and SOFA. This may be related to the clinical setting; our patients, indeed, were hospitalized in a medical ward and outside the ICU.

Furthermore, the best values of NLR, CRP, PCT, and MR-proADM for a diagnosis of sepsis were lower than the values reported from previous studies (10, 5 mg/dL, 0.5 ng/mL, and 1.5 nmol/L respectively) [5,25,26,28,30,31,32,33,34,35,36,37,38,39,40,41,42,43,44]. This may be related to a prompt laboratory evaluation performed immediately after the suspicion of sepsis. These biomarkers, indeed, have a turnaround time of less than an hour for complete blood count and one hour for CRP, PCT, and MR-proADM. A prompt availability of these biomarkers may reduce the delay between the diagnosis of sepsis and the administration of an effective treatment.

As for sepsis diagnosis, NLR values of 9.05 showed a good role in prognostic stratification in terms of 90-day mortality. This is increased by its combination with both MR-proADM (PPV of 52% and NPV of 50%) and clinical scores of sepsis such as SIRS (PPV of 95% and NPV of 86%), q-SOFA (PPV of 96% and NPV of 88%), and SOFA (PPV of 94.6% and NPV of 55.7%).

Knowing that, the shorter the time between clinical presentation and diagnosis, the better the patients’ prognosis, NLR may ameliorate septic patients’ management. This, latter, further increases when the clinical score such as SIRS and qSOFA is used in association with NLR.

The results of our study certainly showed that a prompt and accurate diagnosis of sepsis may be achieved by the use of rapid, cheap, and widely performed biomarkers, as well as in those clinical setting where the use of other biomarkers may be not available or too expensive. Outside the ICU, adding information derived by these biomarkers to clinical score such as SIRS or qSOFA reached a diagnostic accuracy of about 100%.

A limitation of the study is the monocentric enrollment of patients, which should be expanded in the future to be multicentric, thus increasing the number of patients, which is limited to 251 in this first study.

## 5. Conclusions

NLR is a good, rapid, cheap, and widely performed biomarker useful in diagnosis and prognostic stratification of patients with sepsis. The association of NLR with other biomarkers and clinical scores further increases these characteristics. Only the association between clinical signs and several biomarkers may help increase the diagnostic sensibility of sepsis and predict disease severity and mortality. Biomarkers must be performed in supporting a clinical diagnosis. We hope that the use of NLR may improve the management and ameliorate the prognosis of patients with sepsis.

## Figures and Tables

**Table 1:** Baseline patients’ characteristics.

Variables	Patients with Sepsis *N* = 251	Patients without Sepsis *N* = 126	*p*-Value
Age, y	73.0 (65.0, 80.0)	80.0 (68.5, 86.0)	0.001
Male sex, *n* (%)	132 (52.6)	63 (50.4)	0.771
Steroid use, *n* (%)	62 (24.8)	27 (21.6)	0.577
Ongoing chemotherapy, *n* (%)	7 (2.8)	1 (0.8)	0.376
Septic shock, *n* (%)	100 (39.8)	0 (0.0)	<0.001
Smoke history (%)			<0.001
Never	180 (71.7)	55 (44.0)	
Former	61 (24.3)	52 (41.6)	
Current	10 (4.0)	18 (14.4)	
Diabetes mellitus type 2, *n* (%)	56 (22.3)	29 (23.0)	0.981
Cancer, *n* (%)	92 (36.7)	30 (23.8)	0.016
Lung disease, *n* (%)	58 (23.1)	43 (34.1)	0.031
Heart disease, *n* (%)	137 (54.6)	74 (59.2)	0.459
Liver disease, *n* (%)	24 (9.6)	8 (6.3)	0.390
Chronic kidney disease, *n* (%)	73 (29.1)	34 (27.0)	0.760
Chronic cerebrovascular disease, *n* (%)	68 (27.1)	19 (15.1)	0.013
SIRS, median values [IQR]	2 (1, 3)	0 (0, 1)	<0.001
q-SOFA, median values [IQR]	2 (1, 2)	0 (0, 0)	<0.001
SOFA, median values [IQR]	4 (2, 6)	2 (1, 3)	<0.001
NLR, median [IQR]	10.7 (6.3, 18.7)	5.4 (3.7, 7.4)	<0.001
PLR, median [IQR]	228.7 (147.8, 407.9)	219.7 (147.1, 308.2)	0.049
CRP, median [IQR]	107.5 (41.8, 173.7)	8.5 (2.3, 16.5)	<0.001
PCT, median [IQR]	1.2 (0.4, 5.2)	0.1 (0.1, 0.3)	<0.001
MR-proADM, median [IQR]	1.2 (0.8, 1.9)	2.8 (1.8, 4.5)	<0.001
Lenght of stay, median [IQR]	15.0 (11.0, 25.5)	10.0 (7.0, 13.0)	<0.001
ICU admission, *n* (%)	47 (18.7)	0 (0.0)	<0.001
90-day mortality	69 (27.5)	1 (0.8)	<0.001

**Table 2:** Diagnostic role of NLR by ROC curve analysis.

Model	Cut-Off	Sensibility	Specificity	AUC	*p*	PPV	NPV
NLR	7.97	64.26	80.16	0.74	<0.001	86.49	53.18
PLR	370.59	29.3	92.1	0.56	0.037	87.99	39.72
PCR	37.88	78.75	93.51	0.92	<0.0001	95.93	60.46
PCT	0.41	79.6	81.00	0.88	<0.001	89.26	60.67
MR-proADM	1.83	80.1	74.6	0.86	<0.0001	85.51	66.68
SIRS	≥2	67.3	89.7	0.57	<0.001	96.77	43.30
q-SOFA	≥2	51.4	99.2	0.87	<0.001	99.23	50.21
SOFA	≥2	69.7	71.4	0.77	<0.001	82.94	54.21

Area under the curve (AUC); positive predictive value (PPV); negative predictive value (NPV). NLR, neutrophil-to-lymphocyte; PLR, platelet-to-lymphocyte; PCR, C-reactive protein; PCT, procalcitonin; MR-proADM, mid-regional pro-adrenomedullin; SIRS, systemic inflammatory response syndrome; SOFA, sequential organ failure assessment.

**Table 3:** Comparison of the diagnostic role of NLR with other inflammatory markers or clinical scores: positive predictive value (PPV) and negative predictive value (NPV).

Model *	PPV	NPV
NLR	86.49	53.18
PLR	87.99	39.73
PCR	95.93	69.46
PCT	89.26	66.68
MR-proADM	85.52	66.68
SIRS	96.77	43.31
qSOFA	99.23	50.21
SOFA	82.94	54.21
NLR + PLR	96.00	83.00
NLR + PCR	98.70	59.00
NLR + PCT	96.49	61.50
NLR + ADM	95.30	63.30
NLR + SIRS	98.90	81.00
NLR + q-SOFA	99.70	76.00
NLR + SOFA	94.00	73.00
NLR + PLR + SIRS	99.70	94.00
NLR + PLR + q-SOFA	99.90	95.60
NLR + PLR + SOFA	98.30	91.60

* Cut-off values: NLR, 7.97; PLR, 370.59; PCR, 37.88 mg/dL; PCT, 0.41 ng/mL; MRproADM, 1.83 ng/mL; SIRS, q-SOFA, SOFA ≥ 2. NLR, neutrophil-to-lymphocyte; PLR, platelet-to-lymphocyte; PCR, C-reactive protein; PCT, procalcitonin; MR-proADM, mid-regional pro-adrenomedullin; SIRS, systemic inflammatory response syndrome; SOFA, sequential organ failure assessment.

**Table 4:** Role of NLR in predicting 90-day mortality.

Model	Cut-Off	Sensibility	Specificity	AUC	*p*	PPV	NPV
NLR	9.05	69.57	61.44	0.66	<0.001	71.40	89.90
PCR	37.88	83.33	52.35	0.67	<0.001	27.90	93.40
PCT	0.39	90.00	47.00	0.70	<0.001	27.98	95.36
MR-proADM	3.21	76.50	71.40	0.79	<0.001	38.20	92.92
SIRS	≥2	44.29	79.80	0.72	<0.001	33.33	86.26
q-SOFA	≥2	25.70	91.48	0.80	<0.001	40.90	84.20
SOFA	≥2	92.86	52.44	0.82	<0.001	30.80	96.98

Area under the curve (AUC); positive predictive value (PPV); negative predictive value (NPV). NLR, neutrophil-to-lymphocyte; PCR, C-reactive protein; PCT, procalcitonin; MR-proADM, mid-regional pro-adrenomedullin; SIRS, systemic inflammatory response syndrome; SOFA, sequential organ failure assessment.

**Table 5:** Improvement of the prognostic role of NLR with further biomarkers or clinical scores: positive predictive value (PPV) and negative predictive value (NPV) reached by the association of different biomarkers and clinical scores.

Model *	PVV	NPV
NLR	28.9	89.9
NLR + MR-proADM	52.0	50.0
NLR + SIRS	95.0	86.0
NLR + q-SOFA	96.0	88.0
NLR + SOFA	94.6	89.9

* Cut-off values: NLR, 9.05; MRproADM, 3.21 ng/mL; SIRS, q-SOFA, SOFA ≥2. NLR, neutrophil-to-lymphocyte; MR-proADM, mid-regional pro-adrenomedullin; SIRS, systemic inflammatory response syndrome; SOFA, sequential organ failure assessment.

**Table 6:** Multivariate logistic regression model for 90-day mortality.

Model	OR (95% CI)	*p*-Values
NLR	1.002 (0.968 to 1.037)	0.912
PLR	0.999 (0.997 to 1.000)	0.142
MLR	0.952 (0.489 to 1.753)	0.878
CRP	0.998 (0.994 to 1.002)	0.270
PCT	0.989 (0.966 to 1.006)	0.226
MRproADM	1.406 (1.219 to 1.657)	<0.001

## Data Availability

The data presented in this study are available on request from the corresponding author. The data are not publicly available due to their containing information that could compromise the privacy of research participants.

## Abbreviations

AUCs	Areas under the curve
CBC	Complete blood counts
CRP	C-reactive protein
ICU	Intensive care unit
MR-proADM	Mid-regional pro-adrenomedullin
PCR	Polymerase chain reaction
PCT	Procalcitonin
PPV	Positive predictive value
NPV	Negative predictive value
ROC	Receiver operating characteristic
SIRS	Systemic inflammatory response syndrome
SOFA	Sequential sepsis-related organ failure assessment
WBC	White blood cell
